# Reinfestation Sources for Chagas Disease Vector, *Triatoma infestans*, Argentina

**DOI:** 10.3201/eid1207.051445

**Published:** 2006-07

**Authors:** María C. Cecere, Gonzalo M. Vazquez-Prokopec, Ricardo E. Gürtler, Uriel Kitron

**Affiliations:** * Universidad de Buenos Aires, Buenos Aires, Argentina;; † University of Illinois at Urbana-Champaign, Urbana, Illinois, USA

**Keywords:** Chagas disease, Geographic Information Systems, Vector control, Triatoma infestans, Spatial clustering

## Abstract

Reinfestation by *Triatoma infestans* after insecticide spraying has caused elimination efforts in the dry Chaco region to fail repeatedly. The sources and spatial extent that need to be considered to understand the reinfestation pattern and to plan a comprehensive control program were studied in 2 adjacent rural communities in northwestern Argentina from 1993 to 1997. The effects of external, residual, and primary sources on the reinfestation pattern were evaluated by using geographic information systems, satellite imagery, spatial statistics, and 5-year retrospective data for 1,881 sites. The reinfestation process depended on primary internal sources and on surrounding infested communities. In the dry Chaco, successfully reducing the risk for reinfestation in a community depends on treating all communities and isolated sites within 1,500 m of the target community. In addition, during the surveillance phase, spraying all sites within 500 m of new foci will delay reinfestation.

## Abstract 2

Treating all communities within 1,500 m of a target community may reduce reinfestation risk.

*Triatoma infestans*, the main domestic vector of Chagas disease in Latin America, can disperse actively by flying or walking and passively through accidental carriage on humans and their belongings (*1**,**2*). Based mostly on the residual application of pyrethroid insecticides (*3*), an ongoing regional *T. infestans* elimination program has achieved only limited results in the dry Chaco region because of repeated reinfestation. Sources for reinfestation may be residual foci where triatomine bugs survived exposure to insecticides, preexisting foci overlooked by vector control staff, and adjacent infested communities left untreated (*4**–**7*). In northwestern Argentina and Bolivia, peridomestic foci of *T. infestans* detected just 1–3 months after applying pyrethroids were most probably residual foci (*5**,**8**–**10*). In the apparent absence of sylvatic foci of *T. infestans* in northern Argentina (*11*), the appearance of adult *T. infestans* can be explained by active dispersal from foci located in its flight distance (*12**–**14*). This flight distance is well within the range of clustering detected around external (up to 1,500 m) and internal sources (up to 400 m) observed in an earlier study (*7*).

Using geographic information systems, satellite imagery, spatial statistics, and retrospective data collected over 5 years, we identified *T. infestans* sources after community-wide insecticide spraying in an isolated rural community, Amamá, in northwestern Argentina (*7*). One year after spraying, an initial peridomestic focus was detected, and subsequent infestations clustered around it. This clustering suggested that residual spraying with insecticides in the colonized site and all sites in a radius of 450 m is necessary to prevent subsequent propagation of *T. infestans*. However, because the communities under surveillance are surrounded by other infested communities, preventing reinfestation is more complex. As part of a larger project on the ecoepidemiology and control of Chagas disease, we applied spatial tools (*7**,**15*) to analyze spatiotemporal *T. infestans* reinfestation patterns by following a blanket insecticide spraying in 2 adjacent rural communities surrounded by other communities with different histories of infestation. We evaluated the role of various types of *T. infestans* sources on the reinfestation pattern, with the long-term goal of building a metapopulation model of reinfestation.

## Materials and Methods

### Study Area

**Figure 1:**
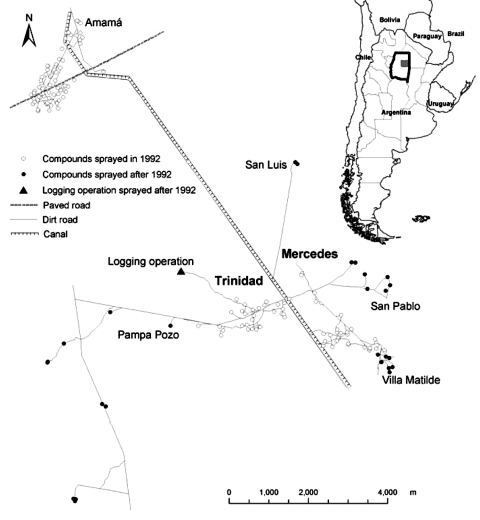
Study area: Trinidad, Mercedes, and neighboring communities, northwestern Argentina. Inset shows location of Moreno Department in Santiago del Estero Province.

Field studies were conducted in the adjacent rural villages of Trinidad and Mercedes (27°12´33´´S, 63°02´10´´W), Santiago del Estero Province, Argentina. These communities were surrounded by other communities with diverse histories of infestation and insecticide spraying. Villa Matilde, San Luis, and San Pablo were close to Mercedes, and Pampa Pozo and a logging operation were close to Trinidad (Figure 1). All communities are located in a semiarid plain where a hardwood forest has been undergoing intensive exploitation. The area and history of infestation by *T. infestans* have been described previously (*5**,**6**,**10*). Communities consisted of 5 to 50 compounds. Most compounds include a domicile made of adobe walls and thatched roofs and a peridomestic area consisting of a patio and 3–8 structures (store rooms, kitchen, corrals, etc.) (*16*) and vary greatly in size. All domiciles were identified with a numbered plaque and mapped in 1992; new and abandoned structures were continuously recorded.

### Mapping and Geospatial Processing

An Ikonos satellite image (Space Imaging, Atlanta, GA, USA) sharpened to 1-m spatial resolution was georeferenced by global positioning system (GPS) (Trimble GeoExplorer II, Trimble Navigation Ltd., Sunnyvale, CA, USA) readings from landmarks in the field. The image and sketch maps from each compound were used to digitize structures that were not located originally with the GPS. The exact location of all structures (sites) was overlaid on the image by using sketch maps from each compound. The entomologic database from Trinidad, Mercedes, and neighboring communities was associated with geographic coordinates (in Universal Transverse Mercator, Zone 20S, WGS1984 datum) of each identified structure by using ArcGIS version 8.1 (Environmental Systems Research Institute, Redlands, CA, USA).

### Field Surveys

In the baseline survey conducted in March 1992, *T. infestans* infested 88% of domiciles and 50% of peridomestic structures and colonized 79% and 38% of them, respectively (*10*). In October 1992, all compounds in Trinidad and Mercedes were sprayed with the pyrethroid deltamethrin (25 mg active ingredient/m^2^) (K-Othrina, Agrevo, San Isidro, Argentina) by the Servicio Nacional de Chagas (NCS). The effectiveness of spraying was then assessed for each site by 2 technicians who spent 10 minutes per compound; all residual foci detected were immediately sprayed in December 1992 (*5*). The surveillance phase included community participation and selective insecticide spraying by NCS in sites with >1 *T. infestans* from 1993 to 1995 and by residents of compounds from 1996 to 2002 (*10*). The adjacent communities of Villa Matilde and Pampa Pozo were sprayed by NCS between October 1993 and May 1994; San Pablo was sprayed by residents in late 1994. The study objectives were explained to residents, and all participants signed an informed consent form.

Each domestic and peridomestic site in Trinidad and Mercedes was searched annually for triatomine insects from October 1993 to November 1997 (*17*). Two skilled insect collectors from NCS searched bedrooms, while another person searched peridomestic structures, for 30 minutes (1 person-hour and 0.5 person-hours, respectively) by using timed manual collections with 0.2% tetramethrin (Icona, Buenos Aires, Argentina) as an irritant agent (the flushing-out method). In peridomestic sites, additional searches for bugs were conducted in May 1995, 1996, and 1997 (0.5 person-hours per peridomestic compound). In May 1993, householders' collections in each compound were initiated by providing a labeled, self-sealing, plastic bag to each household. In addition, from May 1993 to November 1997, domestic sensor boxes (Biosensor, Biocientífica de Avanzada, Buenos Aires, Argentina) placed in bedrooms were inspected semiannually for evidence of infestation. In November 1995, intensive searches for insects by knock-down collections were done in a few domiciles (*17*). All bugs were identified to species and stage (*18*).

### Statistical Analysis

We restricted the analysis of the reinfestation process to 1993 through 1997, when the system was less perturbed by selective insecticide spraying (the effects of which will be presented elsewhere) than thereafter. Infestation and total numbers of *T. infestans* in domestic sites were estimated on the basis of insects collected by flushing out, in sensor boxes, and by householders, and in peridomestic sites by flushing out. Prevalence and abundance of infestations were calculated for all types of peridomestic structures with >1 infested site detected from 1993 to 1997. In this study, cluster refers to an unusual aggregation of sites with high abundance of insects that are grouped together in time and space. Global (weighted K-function) and local (Gi[d]) spatial statistics were used to detect clustering of insects within the study area and to identify epicenters of infestation. The weighted K-function was used to analyze the spatial distribution patterns of abundance of *T. infestans* among all sites in the study area (*19*). A local spatial statistic, such as Gi[d] (*20*), can be used as a focal statistic when the weight of the point being evaluated is not included in the calculation (*7**,**21**,**22*). Gi[d] was used as a focal spatial statistic to measure spatial clustering of *T. infestans* abundance around known and suspected sources of *T. infestans* reinfestation and to calculate the range of distances over which such reinfestation occurred (*7*). Then, clustering occurs as long as Gi[d] values remain significant with increased distance, and peak clustering occurs when Gi[d] is maximized (*20*). When considering >1 site as a potential source, we corrected for multiple comparisons (*23*). Spatial analyses were performed by Point Pattern Analysis software (San Diego State University, San Diego, CA, USA) (*24*).

All sites that were positive after spraying were considered reinfested, including those that were newly infested, those where insects were discovered after intervention that may have survived treatment, and those with insects that had migrated into the trial site after intervention. Reinfestation sources of *T. infestans* were classified as follows: a) within communities, sources were residual if *T. infestans* colonies were detected in December 1992 immediately after the spraying and new otherwise; b) sources were primary if *T. infestans* colonies could not be attributed to other sources and secondary if they could be associated with an earlier primary source; c) internal sources occurred within the community, while external sources were outside the specific community (though they may have been internal to another community).

A compound was invaded when a single adult insect (or very few insects) was found in >1 structure in a given survey. A structure was infested when >1 insect was found in it, and a compound was infested when >1 structure in it was infested. A site was colonized when >1 nymph was found in it, and a compound was colonized when >1 structure in it was colonized.

## Results

**Figure 2:**
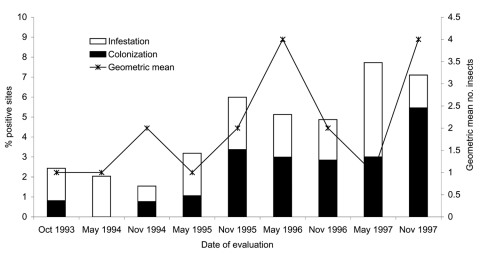
Percentage of infested and colonized sites and number of *Triatoma infestans* per positive site from 1993 to 1997 in Trinidad-Mercedes, Argentina.

The overall prevalence of infestation in Trinidad-Mercedes was <3% through May 1995 and increased to 5%–8% thereafter (Figure 2). Colonization also increased from ≈1% through May 1995 to ≈3% through May 1997 and to >5% in November 1997. The geometric mean number of *T. infestans* per positive site fluctuated from 1 to 4, peaking in May 1996 and November 1997. Of 403 *T. infestans* captured, 248 (62%) were collected from peridomestic sites (Table). Goat corrals had more infested sites and larger *T. infestans* populations than other peridomestic structures.

**Table:** Prevalence of infestation and number of *Triatoma infestans* insects by type of structure in Trinidad-Mercedes, Argentina, October 1993 to November 1997

Structure	No. sites inspected	No. positive sites (%)	Geometric mean no. bugs per infested site	No. *T. infestans*
Domicile	387	44 (11)	1.9	155
Goat corral	296	21 (7)	1.7	142
Kitchen or storeroom	248	15 (6)	2.4	78
Pig corral	239	3 (1)	1.6	15
Chicken coop	43	1 (2)	1	1
Tree (with or without chickens)	308	3 (1)	1	3
Other*	360	1 (0.3)	9	9
Total	1,881	88 (5)	2.2	403

*Included sheds with only a roof, bathrooms, cow and horse corrals, chicken roosts, wood piles, and small granaries. Of these, only 1 small granary was infested.

**Figure 3:**
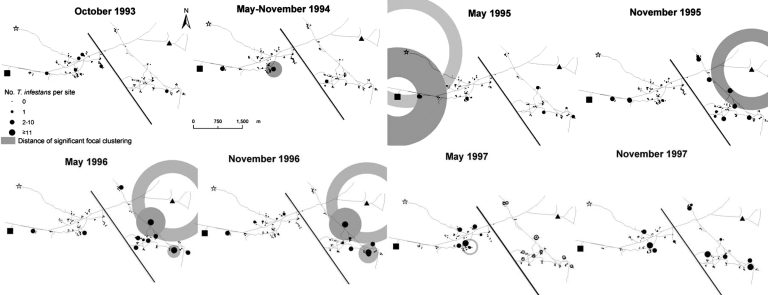
Abundance of *Triatoma infestans* in domestic and peridomestic sites: focal clustering distances of insect abundance around residual foci, primary and secondary sources, and external sources from 1993 to 1997 in Trinidad-Mercedes, Argentina. ¶, logging operation; n, peridomestic structures in Pampa Pozo; p, peridomestic structures in San Pablo.

The spatio-temporal reinfestation process varied between Trinidad and Mercedes (Figure 3). In Trinidad, ≈1.5 year after spraying (February 1994), the residents of 1 compound caught 25 bugs in a chicken coop. By November 1994, one domicile and 3 peridomestic sites (including a small granary) around this chicken coop were infested, and by May 1996, another colony was detected in a goat corral at the same compound. In western Trinidad, in November 1995, one colony was detected east of Pampa Pozo and south of the logging operation. Five years after spraying (1997), the number of infested sites and insects peaked; infestation clustered up to 600 m around a goat corral that hosted the largest colony detected after the 1992 spraying.

In Mercedes, in May 1995, only 2 adults (1 from each of 2 domiciles) and 1 nymph in a storeroom were captured. In November 1995, this storeroom was colonized, and adult insects were captured in the corresponding domicile. The infested site nearest to this storeroom was in the small community of San Pablo. Three years after spraying, insect populations were dispersed all over Mercedes, and by May 1996, the abundance of *T. infestans* per site was higher than ever.

### Residual Foci of *T. infestans*

In Trinidad, 2 residual foci were detected in December 1992, but significant (Gi[d] >2.94, p = 0.05) clustering was detected only in May 1995 around 1 of them and only up to 50 m. Since the effects of this focus overlapped with the effects of the logging operation that was active from 1994 to 1996, this residual focus does not appear to be an independent source of *T. infestans*.

In Mercedes, 2 residual foci were detected in December 1992. Only around 1 of them, a storeroom, did we detect significant (Gi[d] >2.94, p = 0.05) clustering up to 100 m in May 1996, increasing to 250 m in November 1996 (Figure 3). This residual focus was not a likely source of *T. infestans* in 1996, given the time since this source was sprayed in 1992. A primary source detected in 1995 (with which the clustering effect of the residual focus overlapped) provided a more likely source for reinfestation in 1996.

### Primary and Secondary Sources

In Trinidad, a chicken coop was a primary source of reinfestation in May 1994, with substantial focal clustering of insects up to 300 m around it in November 1994 and up to 200 m in May 1997 (Figure 3). Clustering at 300 m was also observed in May 1997 around a granary found to be colonized in November 1994, only 13 m from this primary source. In May 1997, several infested sites were detected in the influence area of the primary and secondary sources; the largest colony was associated with these sources, a goat corral in the center of Trinidad.

In Mercedes, a storeroom was considered a primary source of *T. infestans* in May 1995, when only 1 fifth-instar nymph was collected from it, and in November 1995 when it was found to be colonized and immediately sprayed. Significant focal clustering was registered up to 450 m around this storeroom in May 1996 and up to 500 m in November 1996 (Gi[d] >2.88, p<0.05) (Figure 3). This site was the nearest neighbor (900 m) to a compound in San Pablo infested with *T. infestans* in May 1997 and was believed to have been infested earlier. In February 1994, San Pablo was sprayed with residual insecticides by residents. Since San Pablo was not treated by professional spraying teams and because 1 of its compounds contained a dense colony, we considered it a potential external source of *T. infestans* for Mercedes until 1997.

### External Sources

In Trinidad, 3 external sources of *T. infestans* were tested as potential sources for reinfestation. A small logging operation (Figure 1 and Figure 3) in the northwestern extreme of Trinidad, 1,400 m from the nearest compound in Trinidad and overlooked during the 1992 spraying campaign, was found to be infested 2 years after spraying (1994) and remained infested until November 1996, when it was sprayed. Significant (Gi[d] >2.94, p<0.05) clustering around this site was registered at 1,450–1,700 m in May 1995 (Figure 3). The 5 compounds of the Pampa Pozo community were sprayed 1 year after blanket spraying of Trinidad and Mercedes. Three of the 5 compounds were infested before spraying, and the closest to Trinidad (650 m) was found to be colonized before being sprayed in late 1993. This compound was tested as a potential source of insects, and significant clustering (Gi[d] >2.94, p<0.05) was registered around it from 700 to 1,500 m in May 1995. Thus, the adult invasion registered from November 1994 to 1996 and several infestations in western Trinidad appear to have occurred while a stable focus in the logging operation and the more temporary focus at Pampa Pozo were present.

A compound in San Pablo that was infested in May 1997 and suspected of having been infested earlier was analyzed as an external source of *T. infestans* to Mercedes. A significant (Gi[d] >2.94) clustering at 950–1,450 m in November 1995, and at 950–1,200 m in 1996, was registered around this site (Figure 3). The nearest infested compound of Villa Matilde in October 1993, close to the southeastern extreme of Mercedes, was infested with only adults in November 1995 and May 1996 and was not a likely source of reinfestation.

We also considered infested sites at each of the 2 communities as potential sources for reinfestation in the neighboring community. None of the infested sites in Trinidad was found to have contributed to the reinfestation of Mercedes, nor did the Mercedes sites appear to have contributed to the reinfestation of Trinidad.

In addition to active dispersal, 2 large triatomine colonies, which contained most instars, were detected in domiciles in Mercedes. According to householders' reports, we attribute these colonies to passive transport of *T. infestans* in bags and furniture brought from another logging operation ≈2,600 m away. A search of these belongings turned up numerous nymphs and adult bugs.

## Discussion

The reinfestation dynamics of *T. infestans* in rural areas are heterogeneous in space and time and are a function of processes operating both within and between communities. Because control actions were not applied simultaneously throughout the target area of Trinidad and Mercedes, neighboring communities were external sources of reinfestation, while several peridomestic sites within the 2 communities became internal sources of reinfestation. In Mercedes, reinfestation was driven by a residual focus in 1992 and by a suspected external source in 1993, from which a primary internal source may have originated in 1995. In Trinidad, an internal source (a chicken coop in 1994) and 2 external sources (in 1993) were detected.

Residual foci were detected both in Trinidad and Mercedes, even when the insecticide spraying was performed by professional staff. Domestic residual foci were rare because pyrethroid insecticides have long-lasting residual effects indoors (*25**,**26*) but wane rapidly in peridomestic structures (*27*). Peridomestic residual foci are typically wooden structures with much of their surface exposed to extreme weather conditions and are difficult to spray adequately, as was also noted in the residual foci detected in Amamá in 1992 (*5**,**7*). The residual foci in Trinidad-Mercedes were not primary sources and acted only at relatively short distances (<250 m) within a 30- to 48-month lag. Conversely, in the isolated Amamá, all reinfestation was driven by a residual focus (a pig corral) that became a primary source (*7*).

The effects of primary sources on reinfestation in Trinidad and Mercedes were similar, acting within a 6- to 30-month time lag and within a spatial range of 500 m. Primary sources that developed after blanket spraying produced more sites with high numbers of insects than did residual foci. The spatial range of infestation was notably similar to that registered previously in Amamá (*7*). Thus, primary sources appeared to act similarly in space and time on different types of landscape and arrangements of compounds and on areas with different histories of *T. infestans* infestation. Primary sources also appeared to have originated from external sources or residual foci, at least in our study area. In Mercedes, the primary source probably came from an external source, and in Trinidad it might have been a residual focus that was not detected by flushing-out searches after spraying in 1992 and 1993.

The large insect abundance found in February 1994 in a chicken coop, considered a primary source of reinfestation in Trinidad, indicated that the colony was founded >2 years previously (*28*). This source was probably originally a residual focus that then became a primary source, as with a pig corral in Amamá (*7*). The closer an external source is to the target community, the higher the risk that primary sources will appear in the community. The suspected external source in San Pablo, 600 m from Mercedes, apparently produced a primary focus, while the farthest source, 8 km away in Amamá, was not associated with reinfestation in Trinidad-Mercedes. Other external sources with persistent infestations (the logging operation and Pampa Pozo), located between 0.9 and 1.5 km away, did not produce any primary sources in Trinidad, but frequent findings of adult insects and colonized sites in western Trinidad can be attributed to them. The logging operation was more distant but lasted longer as a source (until it was sprayed in late 1996) and affected a wider area than the Pampa Pozo source that was sprayed earlier (in 1993). Thus, external sources had asynchronous dynamics with respect to internal sources, and their effects varied according to the distance from the target community and the history of infestation.

The primary sources for each community did not serve as external sources for each other, although they were only ≈500 m apart. In part, this lack of effect may be explained by the proportion of suitable habitat surrounding each source and the degree of spatial heterogeneity. Studies of mosquito vectors showed less dispersal of *Aedes aegypti* in areas where compounds were clustered than in areas where they were farther apart, and mosquito vectors tended to be spatially clustered at the household level in rural habitats with abundant human hosts and oviposition sites (*29**,**30*). In our study, internal sources were surrounded by more suitable sites for *T. infestans* than external sources, and the shorter distances between source and target increased the probability that insects would establish a new colony. Furthermore, the canal with running water between Trinidad and Mercedes may have been a barrier to *T. infestans* flight dispersal in each direction. A similar situation was found in Amamá where the northern infestation source was considered independent of the southern source, and the 2 were separated by a canal (*7*).

In addition to active dispersal, passive transport of *T. infestans* in workers' belongings provided an additional means of introducing bugs into communities. The weak local rural economy and unstable occupations of migrant workers enhanced this phenomenon. Contiguity and communication between more distant communities need to be considered for vector control programs in light of passive transport of *T. infestans*.

Our results suggest that control vector programs should cover potential external sources around the target community, at least up to 1,500 m, to reduce adult insect invasion; define the minimum control unit of *T. infestans* to increase cost-effectiveness of chemical control actions; and plan surveillance on the basis of residual spraying of recolonized sites and all sites within 450–500 m to prevent the subsequent propagation of *T. infestans*. Future work will aim to improve our understanding of the *T. infestans* reinfestation process under different regional conditions.
